# Modulating protein activity using tethered ligands with mutually exclusive binding sites

**DOI:** 10.1038/ncomms8830

**Published:** 2015-07-22

**Authors:** Alberto Schena, Rudolf Griss, Kai Johnsson

**Affiliations:** 1 École Polytechnique Fédérale de Lausanne, Institute of Chemical Sciences and Engineering, Avenue Forel 2, EPFL SB ISIC LIP BCH-4303, CH-1015 Lausanne, Switzerland; 2 École Polytechnique Fédérale de Lausanne, Institute of Bioengineering, CH-1015 Lausanne, Switzerland; 3 National Centre of Competence in Research in Chemical Biology, CH-1015 Lausanne, Switzerland

## Abstract

The possibility to design proteins whose activities can be switched on and off by unrelated effector molecules would enable applications in various research areas, ranging from biosensing to synthetic biology. We describe here a general method to modulate the activity of a protein in response to the concentration of a specific effector. The approach is based on synthetic ligands that possess two mutually exclusive binding sites, one for the protein of interest and one for the effector. Tethering such a ligand to the protein of interest results in an intramolecular ligand–protein interaction that can be disrupted through the presence of the effector. Specifically, we introduce a luciferase controlled by another protein, a human carbonic anhydrase whose activity can be controlled by proteins or small molecules *in vitro* and on living cells, and novel fluorescent and bioluminescent biosensors.

## Abstract (web summary)

Designing proteins whose activities can be switched on and off by effector molecules is a central challenge in protein engineering. Here, the authors use tethered chemical ligands with two mutually exclusive binding sites as a general method to modulate protein activity in response to specific effectors.

## Graphical abstract

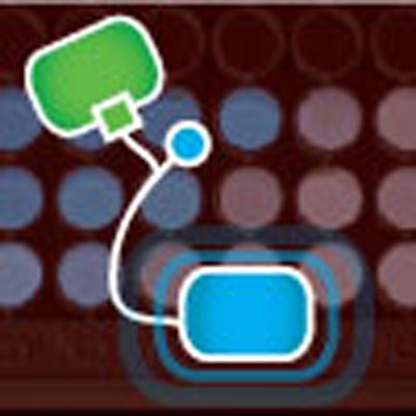

Allosteric proteins act as molecular switches in which binding of a molecule to a site different from the active site changes the conformation of the protein and its underlying activity. Such proteins are fundamental for the regulation of most natural signalling processes. Designing new allosteric proteins is a formidable test for our understanding of protein function and such designer proteins can find applications in synthetic biology and biosensing^1^,^2^,^3^,^4^. For example, the most popular fluorescence-based biosensor for live-cell imaging is based on an engineered fluorescent protein allosterically regulated by calcium ions^5^.

The design of novel allosteric proteins is usually based on the insertion of a pre-existing allosteric protein domain into another protein; binding of the allosteric modulator then changes the conformation of the allosteric domain and of the protein in which it is inserted^6^,^7^,^8^. However, the generality of the approach is limited as it relies on pre-existing allosteric proteins and modulators. In addition, the identification of an appropriate insertion site is difficult. Alternative approaches for regulating protein activity have been developed. For example, enzymatic activities have been regulated by tethering an inhibitor to an enzyme through a single-stranded oligonucleotide; binding of a complementary DNA sequence prevents an intramolecular inhibition of the enzyme^9^,^10^. However, in its present form the approach is limited to DNA as an effector molecule. In another approach, the activity of an enzyme is regulated by introducing new binding sites in close proximity to its active site^11^. This principle is the basis for the enzyme multiplied immunoassay technique—the first homogeneous immunoassays—which are still commonly used in diagnostics today^12^,^13^. However, not every enzyme lends itself to such modifications of its active site. In short, alternative design principles for the generation of proteins that can exist in two different states that are energetically similar but differ in activity are needed.

We introduce here a conceptually novel approach to regulate protein activity. As with allosteric proteins, the activity of the reporter is modulated by an external effector. However, the modulation is not based on a conformational change within the reporter protein but on the steric displacement of a ligand in a larger semisynthetic protein construct. This is achieved by generating synthetic ligands that possess two mutually exclusive binding sites. We demonstrate the potential of the approach by generating a novel bioluminogenic protein as well as bioluminescent and fluorescent biosensors for applications *in vitro* and in live cells.

## Results

### Effector-modulated reporters

We have previously introduced fluorescent and bioluminescent sensor proteins that are based on the competition of a tethered ligand with an analyte for a common binding site^14^,^15^,^16^,^17^,^18^. The binding and unbinding of the tethered ligand leads to a change in fluorescence resonance energy transfer (FRET) or bioluminescence resonance energy transfer (BRET) efficiency and can be used as readout for the concentration of the competing analyte. We speculated that adding a second synthetic ligand that binds to its target in a mutually exclusive manner with respect to the first tethered ligand could allow the modulation of protein activity by an unrelated effector. In this approach, the effector binds to one of the two synthetic ligands, making the other one unavailable for interactions with the protein to which the synthetic ligands are tethered. The task of modulating protein activities is thus reduced to either the modification of an existing ligand–protein interaction or the design of a new one and opens up the possibility to transform regular proteins into effector-modulated reporters resembling allosteric proteins. In the following, we will abbreviate our approach as chemical ligand-associated steric hindrance (CLASH).

### Control of a luciferase by an exogenous effector

**Figure 1:**
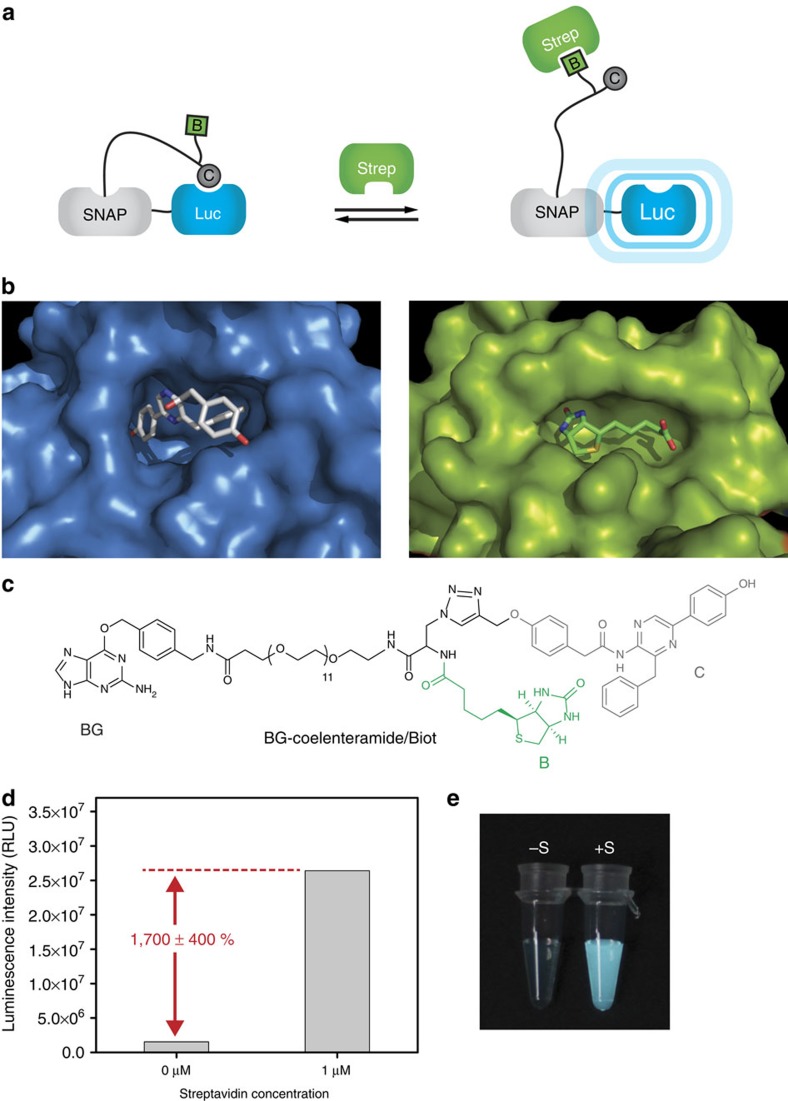
Control of a luciferase. (**a**) Schematic principle of the regulation of a luciferase by an exogenous effector: the dual ligand coelenteramide/biotin can bind to luciferase (Luc) or streptavidin (Strep) separately but not to both at the same time. Binding of streptavidin to the secondary ligand biotin (B) makes the tethered coelenteramide (C) unbind from the luciferase active site, leading to a change in bioluminescence. (**b**) Crystal structures of the active sites of *Renilla* luciferase with the inhibitor coelenteramide (blue, PDB ID 2PSJ) and of streptavidin bound to biotin (green, PDB ID 3RY2). (**c**) Structure of the dual ligand labelling molecule. (**d**) Luminescence intensity of 100 μl of 25 nM CLASH-Strep/Luc mixed with 2.5 μg ml^−1^ of the substrate coelenterazine in absence and in presence of 1 μM streptavidin. (**e**) Picture of the same solutions in transparent tubes, taken with a Canon 600D camera.

Controlling the light-emitting properties of luciferases through molecules of interest is an attractive approach to generate powerful biosensors. Luciferases allosterically controlled by either calcium ions or cyclic AMP have been generated by inserting naturally occurring allosteric protein domains^19^,^20^, but to the best of our knowledge no luciferases have been designed that are regulated by other proteins. We thus exploited CLASH to transform the luciferase from *Renilla reniformis* into one that is activated by an effector protein. For first proof-of-principle experiments, we chose the protein streptavidin as the effector, since its natural ligand biotin can be readily synthetically derivatized. We synthesized a dual ligand containing the luciferase inhibitor coelenteramide^21^ and biotin in such close proximity that simultaneous binding to the luciferase and to streptavidin should be disfavoured (Fig. 1a,b). Using SNAP-tag technology^22^, we chemically linked the dual ligand to the *R. reniformis* luciferase, generating CLASH-Strep/Luc (Fig. 1c). In the absence of streptavidin, the tethered coelenteramide inhibits the luciferase, resulting in a dark state. On addition of streptavidin, the light intensity increased in a concentration-dependent manner: binding of streptavidin to the biotin moiety of the synthetic ligand forces coelenteramide to dissociate from the luciferase, leading to an active luciferase. The light intensity increased by 1,700% in the presence of streptavidin, permitting streptavidin detection by naked eye (Fig. 1d,e). The marked increase in light emission in response to an analyte makes the approach an attractive starting point for generating biosensors for various proteins. This then would require the replacement of biotin in our synthetic ligand with an appropriate binder to the protein of interest.

### Control of human carbonic anhydrase

**Figure 2:**
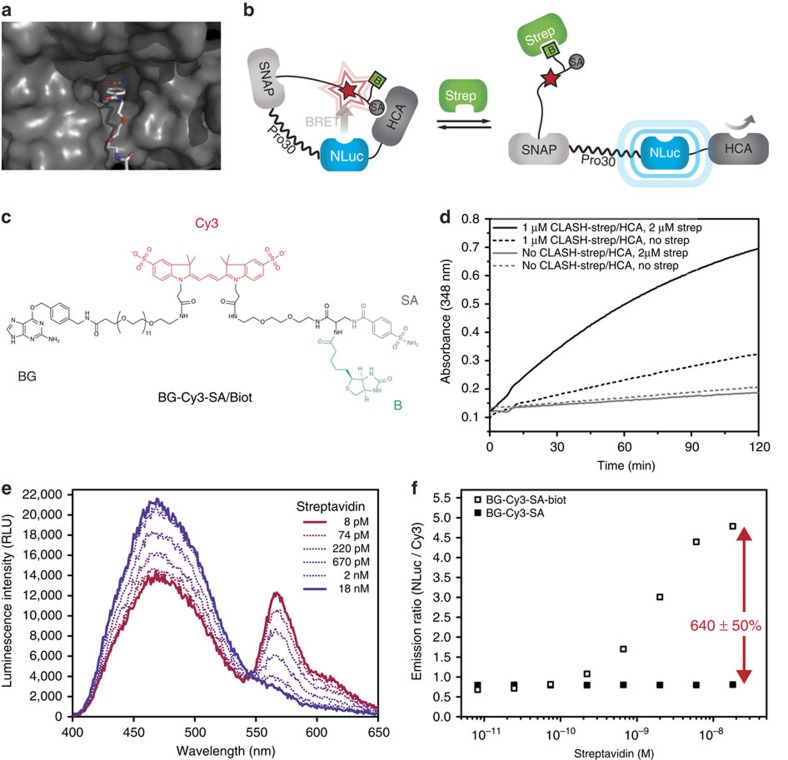
Control of HCA by streptavidin. (**a**) Crystal structure of the active site of HCA bound to a benzenesulfonamide (PDB ID 1CNW). (**b**) Modulation of HCA activity in CLASH-Strep/HCA. A fusion protein of SNAP-tag, a 30-proline linker, NanoLuc Luciferase (NLuc) and HCA and is labelled with a synthetic molecule containing a fluorophore (red star) and the two intramolecular ligands benzenesulfonamide (SA) and biotin (B). Binding of streptavidin (Strep) to the tethered B displaces SA from HCA, leading both to a conformational change that can be seen by a decrease in BRET efficiency, and to an increase of enzymatic activity. (**c**) Chemical structure of the labelling compound. (**d**) Enzymatic activity of HCA and followed by measuring absorbance at 348 nm. Streptavidin (Strep) acts as an effector of the hydrolytic activity of HCA: in the absence of Strep, SA binds to and inhibits HCA (black dashed line), while on addition of saturation concentrations of Strep, the catalytic activity increases (black solid line). (**e**) Emission spectra of 10 nM CLASH-Strep/HCA at increasing streptavidin concentrations. (**f**) Titration of 10 nM CLASH-Strep/HCA with streptavidin. As a control, the synthetic ligand BG-Cy3-SA lacking the tethered biotin is used.

To demonstrate the generality of CLASH, we applied it to regulate the activity of the enzyme human carbonic anhydrase II (HCA). Furthermore, we wanted to translate the activity modulation into a signal compatible with biosensing. We synthesized a dual ligand in which the HCA inhibitor benzenesulfonamide and a biotin moiety are connected in a way that binding to their respective protein receptors would be mutually exclusive (Fig. 2a–c) and used again streptavidin as the effector. The choice of the linker composition and length in the labelling molecule was based on the crystal structure of the receptor proteins. To couple HCA activity switching to a readout compatible with biosensing, we expressed HCA as a fusion protein with the luciferase NanoLuc, a 30-proline linker (PP30) and SNAP-tag, following the design principles of LUCIDs—our previously described class of BRET-based biosensors^18^. The SNAP-PP30-NLuc-HCA fusion protein was labelled with the synthetic ligand also containing the fluorophore Cy3 for the BRET readout. The resulting semisynthetic protein CLASH-Strep/HCA (Fig. 2b) is expected to show an increase in HCA activity and display a change in BRET efficiency between NanoLuc and Cy3 when the tethered ligand is displaced from HCA through binding of the effector streptavidin. The interaction between streptavidin and biotin has a *K*_d_ in the femtomolar range; since this is much lower than the concentration of CLASH-Strept/HCA in the assay, the transition of the titration curve occurs around the concentration of the CLASH construct.

To assess the control of HCA activity in CLASH-Strep/HCA, we performed a standard HCA activity assay based on the catalysis of a chromogenic reaction^23^. In the absence of streptavidin, the construct showed low HCA activity, but on addition of saturating concentrations of streptavidin, the catalytic activity was restored (Fig. 2d). This is in agreement with an inhibition of the HCA activity through the intramolecular benzenesulfonamide in the absence of streptavidin, while on binding of streptavidin to the dual ligand, the benzenesulfonamide is displaced from HCA and the hydrolytic activity is restored. We also tested CLASH-Strep/HCA for BRET efficiency changes at increasing concentrations of streptavidin. We measured an overall BRET ratio change of 640±50% (Fig. 2e,f). The same ratio change was measured when opening CLASH-Strep/HCA by addition of high concentrations of the HCA inhibitor benzenesulfonamide (Supplementary Fig. 1), confirming that the binding of streptavidin to biotin indeed leads to the unbinding of benzenesulfonamide from HCA. When the synthetic ligand BG-Cy3-SA that lacks the biotin moiety was used to label the protein, no response to streptavidin was observed (Fig. 2f).

**Figure 3:**
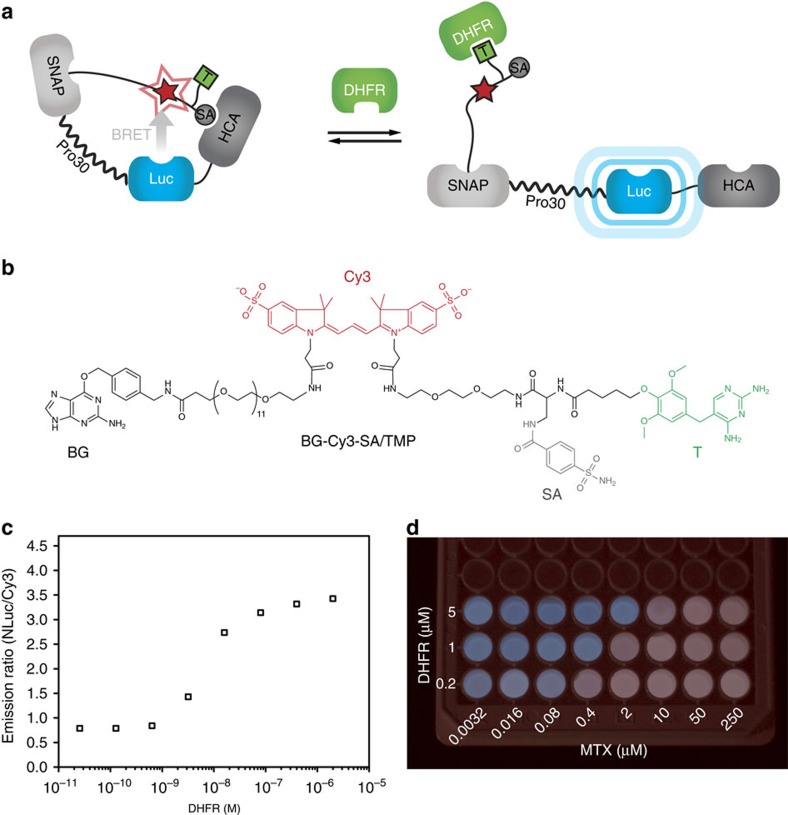
Modulation of HCA by DHFR and DHFR ligands. Cartoon of CLASH-DHFR/HCA for control of HCA with DHFR: DHFR acts as an effector by binding to the tethered trimethoprim (T) and leading to the unbinding of benzenesulfonamide (SA) from HCA. (**b**) Chemical structure of the labelling molecule. (**c**) The opening of the biosensor can be detected by the increase in emission ratio on addition of DHFR. (**d**) Titrations with the anticancer agent methotrexate (MTX) at three different free DHFR concentrations show that the colour of the luminescence emission changes at the threshold concentration. Picture taken with a Canon 600D camera. The colour balance was modified to give a clear difference between the blue and red colours.

To demonstrate the modularity of our approach, we took the same HCA fusion protein as above, but used the enzyme dihydrofolate reductase (DHFR) as the effector. Towards this end, we labelled SNAP-PP30-NLuc-HCA with a synthetic ligand that consists of the DHFR-ligand trimethoprim and benzenesulfonamide, generating CLASH-DHFR/HCA (Fig. 3a,b). In the absence of DHFR, the benzenesulfonamide occupies the active site of HCA and CLASH-DHFR/HCA is in a closed conformation, as shown by the high BRET efficiency (Fig. 3c). In the presence of sufficient concentrations of DHFR, the binding of the tethered sulfonamide to the active site of HCA is prevented and CLASH-DHFR/HCA adopts an open conformation, as demonstrated by its low BRET efficiency.

By mixing CLASH-DHFR/HCA with a defined concentration of free DHFR, CLASH-DHFR/HCA can be easily transformed into a biosensor for DHFR ligands such as the anticancer agent methotrexate (MTX), commonly monitored in hospital patients^24^. DHFR brings the sensor molecule into its open conformation and can be displaced by MTX. If the concentration of MTX is lower than the concentration of free DHFR, the drug is quantitatively bound to DHFR, while above this threshold the excess of free MTX disrupts the interaction between DHFR and tethered trimethoprim, thereby bringing CLASH-DHFR/HCA to its high-BRET state. At the stage where all free DHFR is bound to MTX, only a small relative increase in MTX concentration is necessary to displace the DHFR bound to the sensor molecule, giving a sharp transition. The transition can be easily seen with the naked eye and can be shifted by simply changing the concentration of free DHFR (Fig. 3d; Supplementary Fig. 2).

While a similar system could also be obtained using the previously introduced DHFR-based LUCID for MTX^18^, the present approach opens up the possibility to construct ratiometric BRET sensors with binding proteins that are geometrically not well suited for LUCID sensors. For example, an antibody can replace the free DHFR and one of the two binding sites of our synthetic ligand can be the antigen. The plethora of commercially available antibodies would allow transforming CLASH-antigen/HCA into biosensors for a multitude of analytes. We demonstrated this principle by titrating CLASH-Strep/HCA with anti-biotin monoclonal antibody. In accordance with the CLASH principle, at increasing concentrations of the antibody the BRET efficiency decreases (Supplementary Fig. 3).

### Use of CLASH in live-cell imaging

Labelling of SNAP-tag fusion proteins can be achieved *in vitro* and *in vivo*, including whole animals^25^. While the low permeability of the synthetic ligands we describe here would make intracellular applications difficult, the selectivity of the labelling reaction opens up the possibility to generate effector-regulated proteins on the surface of living cells, either to control protein activities or for sensing applications. To demonstrate this possibility, we aimed at displaying a CLASH-AChE/HCA on HEK293 cells in which the activity of HCA is regulated by compounds targeting acetylcholine esterase (AChE). Controlling the activity of an enzyme such as HCA that is part of a family of closely related enzymes is difficult since specific inhibitors are rarely available. Achieving control with an unrelated compound that does not interact with any other member of such family is of interest for mechanistic studies. As compounds targeting AChE are used as drugs, pesticides or nerve agents, our CLASH-AChE/HCA could in addition be used for biosensing applications. We have previously reported a FRET-based biosensor for detecting such compounds, ACh-SNIFIT^26^, which however suffered from relatively small ratio changes^16^.

**Figure 4:**
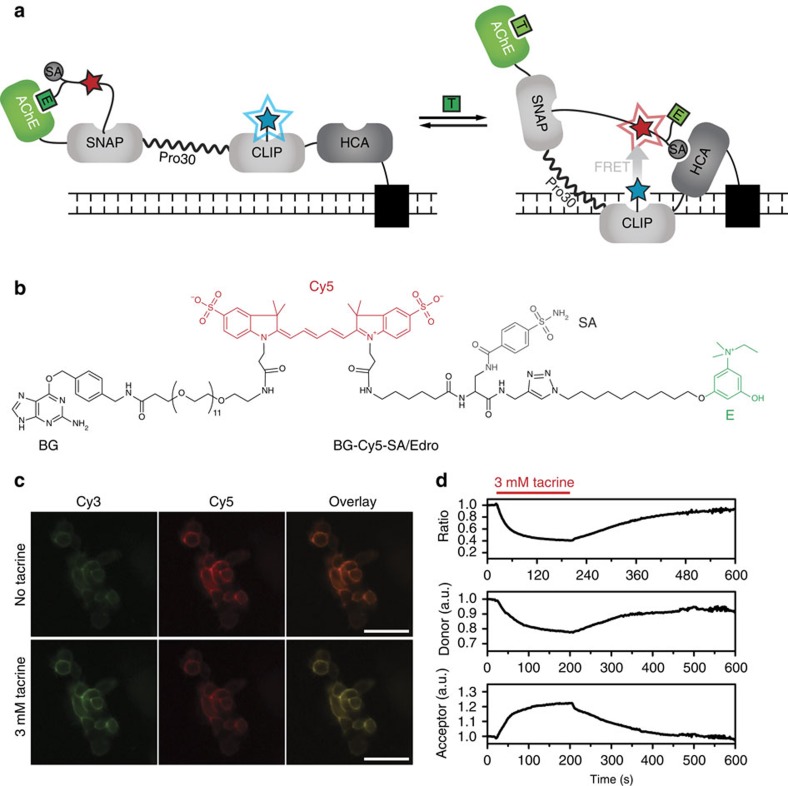
Modulation of HCA by tacrine on living cells. (**a**) Cartoon of CLASH-AChE/HCA. The acetylcholine esterase (AChE) inhibitor tacrine (T) displaces edrophonium (E) from AChE and benzenesulfonamide (SA) can bind to HCA inducing an increase in FRET efficiency. (**b**) Structure of the labelling compound. (**c**) Fluorescence micrographs of HEK293 cells displaying CLASH-AChE/HCA on their surface. In the absence of tacrine Cy3 emission is more intense, while on addition tacrine Cy5 emission increases; scale bars, 50 μm. (**d**) Changes observed in FRET ratio, donor (Cy3) and acceptor (Cy5) emission on perfusion of HEK293 cells displaying CLASH-AChE/HCA on their surface with 3 mM tacrine.

To create CLASH-AChE/HCA, AChE was fused to the N-terminus of an HCA-based SNIFIT (Fig. 4a). We targeted the construct to the outer cell membrane of transiently transfected HEK293 cells. The cells were labelled with a dual ligand containing benzenesulfonamide as primary ligand and edrophonium, an inhibitor of AChE^26^, as secondary ligand (Fig. 4b). In the absence of free AChE inhibitors, the stronger interaction of edrophonium with AChE prevents the binding of the primary ligand to HCA, keeping the sensor in its low-FRET state. When the AChE inhibitor tacrine, a drug used in the past to treat Alzheimer's disease, was perfused on living cells expressing CLASH-AChE/HCA, tacrine displaced the secondary ligand from AChE, allowing the benzenesulfonamide to bind HCA and leading to high FRET efficiency of the sensor (Fig. 4c-d). Importantly, the switching between the two states was fully reversible, and the overall measured FRET ratio change was twofold higher than the original ACh-SNIFIT^26^. In CLASH-AChE/HCA, the relative strength of the two synthetic ligands is such that HCA is active in the absence of the effector targeting AChE and inhibited by its addition (see also Supplementary Discussion for a more detailed discussion of the underlying design principles). Choosing a stronger HCA ligand, it should be equally possible to generate a variant, namely CLASH-HCA/AChE, in which AChE activity is controlled by small benzenesulfonamides. As AChE is a key enzyme for neurotransmission both at the central and at the peripheral nervous systems, such a CLASH-HCA/AChE could find applications as a research tool in neurobiology. For example, inactivating its AChE activity by the addition of a biorthogonal small molecule, for example, a sulfonamide, could help in elucidating the role of cholinergic pathways in neurobiology.

## Discussion

In conclusion, CLASH represents a general concept to control protein function in response to an unrelated effector. The approach allowed us to transform a regular luciferase in a bioluminogenic probe responding with marked increase in emitted light on addition of a regulator. We applied the same principle to control the activity of the enzyme HCA by three completely different ligands, two proteins and a small molecule. The possibility to modulate protein activities on the surface of living cells opens up applications beyond *in vitro* assays. While the feasibility of our approach was demonstrated for a number of unrelated proteins, we would like to reiterate that it is restricted to proteins for which ligands with mutually exclusive binding sites exist or can be designed. Finally, this work highlights how the combination of synthetic chemistry and protein engineering can be exploited to create proteins with entirely new functionalities.

## Methods

### Synthesis of the labelling compounds

Detailed procedures for the synthesis of all compounds and SNAP-tag substrates, their characterization, the titration in multiwell plates and the imaging experiments are given in the Supplementary Methods.

### Cloning and protein expression

All sensor constructs were obtained from the pET51b(+)-based construct SNAP-PP30-NLuc-HCA described in ref. 18 or from pDisplay-based construct SNAP-PP30-CLIP-HCA^16^ using standard cloning techniques. The amino-acid sequences of the described proteins can be found in Supplementary Note 1. The proteins were expressed in the *Escherichia coli* strain Rosetta-gami (DE3). Bacterial cultures in LB or 2 × YT medium were grown at 37 °C to an *OD*_600 nm_ of 0.8, at which point the temperature was lowered to 16 °C, and 0.5 mM isopropyl β-D-thiogalactopyranoside was added. After 16 h, the cells were harvested by centrifugation and lysed by sonication. The cell extracts were cleared by centrifugation and purified in two steps using Ni-NTA (Qiagen) and Strep-Tactin (IBA) according to the suppliers' instructions. The proteins were then transferred into HEPES buffer (50 mM HEPES, pH 7.2, 50 mM NaCl) using Amicon Ultra-0.5 Ultracel-30K centrifugal filters (Millipore). Solutions with proteins containing HCA were supplemented with a twofold molar excess of ZnCl_2_.

### SNAP-tag labelling

The sensor proteins were diluted to 1 μM in HEPES buffer containing 4 μM of the corresponding SNAP-tag substrate. After incubation at room temperature for 1 h, the labelled sensor constructs were purified using Amicon Ultra-0.5 Ultracel-30K centrifugal filters (Millipore), washing three times with 0.4 ml HEPES buffer.

### Luciferase activity assay

The labelled protein SNAP-hRLuc was diluted to a concentration of 50 nM in HEPES buffer with or without 2 μM streptavidin (New England Biolabs). One hundred microlitres were added to the wells of a white nonbinding 96-well plate (Greiner Bio-One). The luciferase activity was then measured on an EnVision Multilabel Reader (PerkinElmer): 100 μl of 5 mg l^−1^ benzyl coelenterazine (NanoLight Technology) was added to the wells using the system's injector and the luminescence intensity was measured after 5 s. Photographs were taken with a Canon 600D digital camera.

### HCA activity assay

HCA hydrolytic activity assays were performed using the standard *p*-nitrophenyl acetate assay^23^: 1 μM of the protein was mixed with 1 mM *p*-nitrophenyl acetate in HEPES buffer. The rates of the HCA catalysed reactions were measured by following the absorbance at 348 nm characteristic of the reaction product using a SPECTRAmax 340 spectrophotometer (Molecular Devices).

### BRET assays

The labelled proteins were diluted to concentrations of 20 nM in 50 μl HEPES buffer containing 0.5 mg ml^−1^ bovine serum albumin and spiked with known concentrations of analyte in white nonbinding 96-well plates (Greiner Bio-One). After incubation at room temperature for 15–30 min, 50 μl Nano-Glo Luciferase Assay Substrate (Promega) diluted 50-fold in Nano-Glo Luciferase Assay Buffer (Promega) was added. Bioluminescence was measured on an EnVision Multilabel Reader (PerkinElmer). The signal was collected using an emission filter for Umbelliferone (wavelength: 460 nm and bandwidth: 25 nm) to record NanoLuc emission and a filter for Cy3 (wavelength: 595 nm and bandwidth: 60 nm) to record Cy3 emission. Emission spectra were measured on an Infinite M1000 spectrofluorometer (Tecan) with a step size of 1 nm, a bandwidth of 10 nm and an integration time of 100 ms. Assays involving DHFR were performed in the presence of 50 or 100 μM NADPH. Biotin monoclonal antibody (BTN.4) was purchased from Fischer Scientific (THERMO SCIENTIFIC PIERCE AB MA5-11251).

### Live-cell FRET imaging

HEK293 cells were grown on polylysine-coated glass coverslips (Ø 15 mm) in DMEM Glutamax medium (Lonza) supplemented with 10% fetal bovine serum (Lonza) and transiently transfected with the appropriate plasmid using Lipofectamine (Invitrogen) according to the manufacturer's protocol. Twenty-four hours after transfection, HEK293 cells were labelled with a solution of 2 μM of the corresponding BG derivative and 10 μM of the corresponding O2-benzylcytosine derivative in Hank's buffered salt solution complemented with 10 mg ml^−1^ bovine serum albumin for 10 min at room temperature. After being labelled, cells were washed four times with Hank's buffered salt solution. Glass coverslips with labelled HEK293 cells were transferred to a Warner imaging chamber (RC-20). Perfusion of the chamber was performed gravity fed at a flow rate of 0.5 ml min^−1^. Time-course experiments of sensor imaging were performed using a Leica LAS AF 7000 widefield microscope equipped with a 40 × plan Apochromat 1.25 numerical aperture oil immersion objective lens. A xenon arc lamp was used for imaging of the HEK293 cells. For each frame, the two channels (donor and FRET) were measured consecutively with an interval of 30 ms between the two emission channels. The following filter sets were used for the FRET ratio imaging: for Cy3/Cy5, excitation at 530 nm (bandwidth 35 nm), emission at 580 nm (bandwidth 40 nm) (Cy3) and at 700 nm (bandwidth 72 nm) (Cy5). If not indicated otherwise, the image size was 293 × 293 μM, and an average of five cells per image was analysed for the intensity ratio plots.

## 

## Additional information

**How to cite this article**: Schena, A. *et al.* Modulating protein activity using tethered ligands with mutually exclusive binding sites. *Nat. Commun.* 6:7830 doi: 10.1038/ncomms8830 (2015).

## Supplementary Material

Supplementary Information Supplementary Figures 1-3, Supplementary Note 1, Supplementary Discussion, Supplementary Methods and Supplementary References
