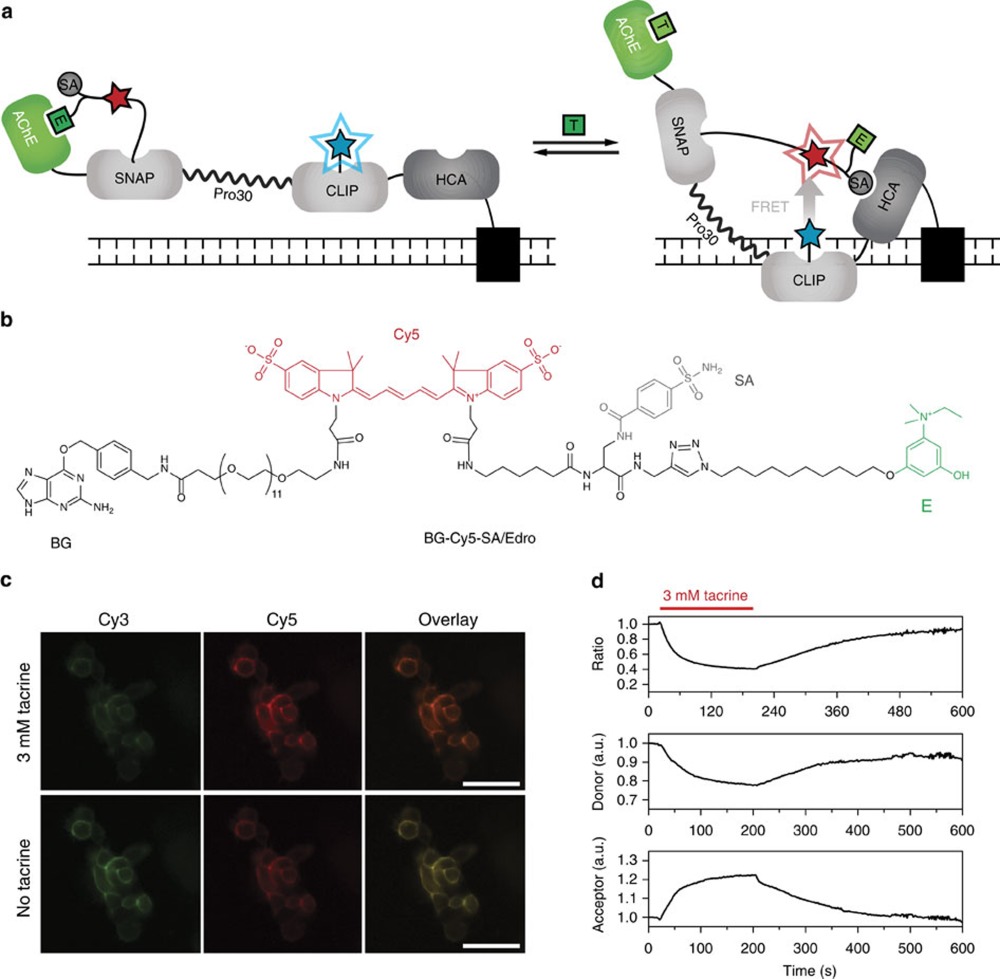# Corrigendum: Modulating protein activity using tethered ligands with mutually exclusive binding sites

**DOI:** 10.1038/ncomms10758

**Published:** 2016-02-15

**Authors:** Alberto Schena, Rudolf Griss, Kai Johnsson

*Nature Communications* 6: Article number: 783010.1038/ncomms8830 (2016); Published 07222015; Updated 02152016

The original version of this article was published with an incorrect label for Fig. 4c. The labels ‘no tacrine' and ‘3 mM tacrine' were associated with the incorrect fluorescence micrographs. The correct version of Fig. 4 appears below.

**Figure 4**