# Supplementary material for: Modulating protein activity using tethered ligands with mutually exclusive binding sites
Source: Nat Commun. 2015 Jul 22;6:7830. doi: 10.1038/ncomms8830 (PMC4525150; doi:10.1038/ncomms8830)
Supplement: Supplementary Information — Supplementary Figures 1-3, Supplementary Note 1, Supplementary Discussion, Supplementary Methods and Supplementary References [file ncomms8830-s1.pdf]

SUPPLEMENTARY FIGURE 1

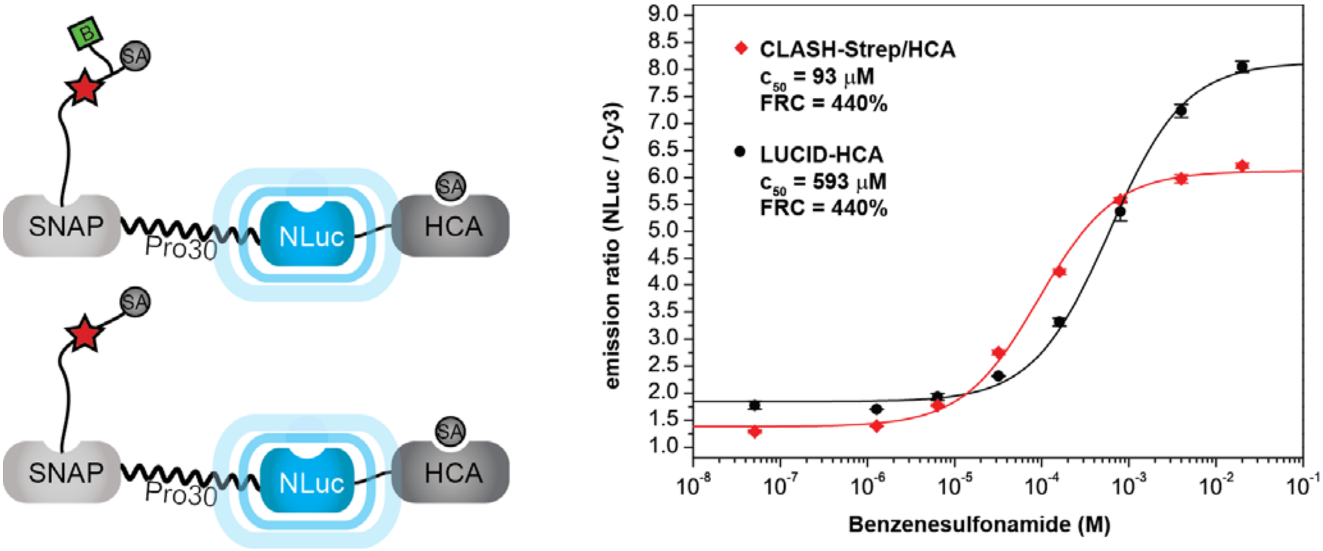

Titrations of CLASH-Strep/HCA with the HCA inhibitor benzenesulfonamide and comparison with the original HCA-based LUCID.

SUPPLEMENTARY FIGURE 2

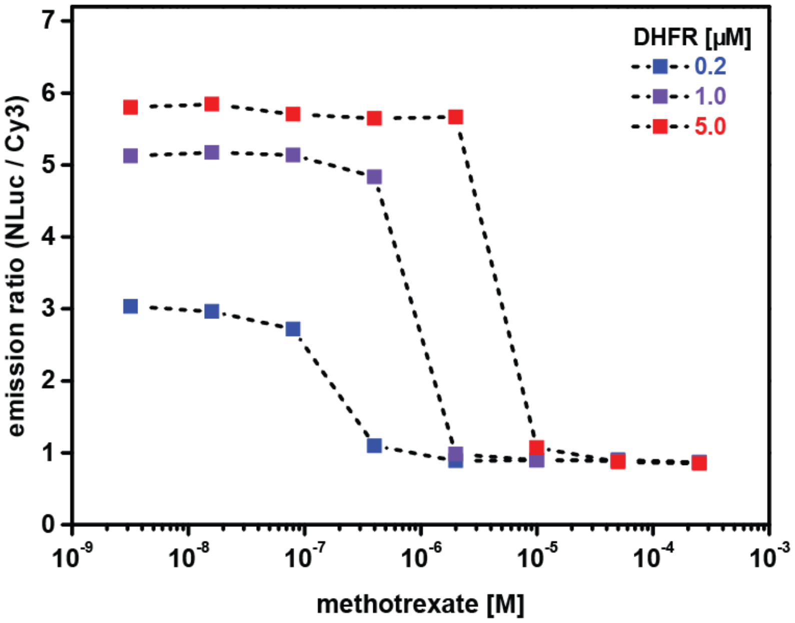

Emission ratios at increasing methotrexate concentrations measured on the 96-well plate shown in Figure 3d.

SUPPLEMENTARY FIGURE 3

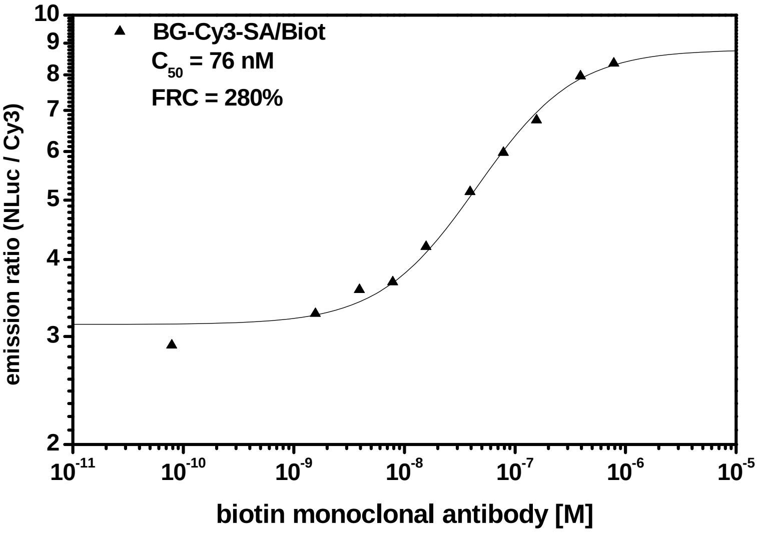

Titration of *CLASH*-Strep/HCA with an anti-biotin monoclonal antibody. Due to the low concentration of the antibody in the commercial stock, the titration was performed in the same buffer in which the antibody was shipped: phosphate buffered saline, pH 7.4, with 0.2% BSA and 0.09% sodium azide. The different buffer and in particular the presence of sodium azide in the buffer explains the difference in BRET-ratios compared to the titration with benzenesulfonamide in Supplementary Fig. 2.

SUPPLEMENTARY NOTE 1: Protein sequences

SNAP-hRLuc

MASWSHPQFEKGADDDDKVPHMDKDCEMKRTTLDSPLGKLELSGCEQGLHEIIFLGKGTSAADAVEVPAPAAVLGGPEP
LMQATAWLNAYFHQPEAIEEFPVPALHHPVFQQESFTRQVLWKLLKVVKFGEVISYSHLAALAGNPAATAAVKTALSGNPVPILIPCHR
VVQGDLDVGGYEGGLAVKEWLLAHEGHRLGKPGLGGRMASKVYDPEQRKRMITGPQWWARCKQMNVLDSFINYYDSEKHAENAV
IFLHGNAASSYLWRHVVPHIEPVARCIIPDLIGMGKSGKSGNGSYRLLDHYKYLTAWFELLNLPKKIIFVGHDWGACLAFHYSYEHQDK
IKAIVHAESVVDVIESWDEWPDIEEDIALIKSEEGEKMVLENNFFVETMLPSKIMRKLEPEEFAAYLEPFKEKGEVRRPTLSWPREIPLV
KGGKPDVVQIVRNYNAYLRASDDLPKMFIESDPGFFSNAIVEGAKKFPNTEFVKVKGLHFSQEDAPDEMGKYIKSFVERVLKNEQGA
PGFSSISAHHHHHHHHHH

SNAP-Pro30-NanoLuc-HCA

MASWSHPQFEKGADDDDKVPHMDKDCEMKRTTLDSPLGKLELSGCEQGLHEIIFLGKGTSAADAVEVPAPAAVLGGPEP
LMQATAWLNAYFHQPEAIEEFPVPALHHPVFQQESFTRQVLWKLLKVVKFGEVISYSHLAALAGNPAATAAVKTALSGNPVPILIPCHR
VVQGDLDVGGYEGGLAVKEWLLAHEGHRLGKPGLGEFPPPPPPPPPPPPPPPPPPPPPPPPPPPPPPGGRMVFTLEDFVGDWRQT
AGYNLDQVLEQGGVSSLFQNLGVSVTPIQRIVLSGENGLKIDIHVIIPYEGLSGDQMGQIEKIFKVVYPVDDHHFKVILHYGTLVIDGVTP
NMIDYFGRPYEGIAVFDGKKITVTGTLWNGNKIIDERLINPDGSLLFRVTINGVTGWRLCERILALEMSHHWGYGKHNGPEHWHKDFPI
AKGERQSPVDIDTHTAKYDPSLKPLSVSYDQATSLRILNNGHAFNVEFDDSQDKAVLKGGPLDGTYRLIQFHFHWGSLDGQGSEHTV
DKKKYAAELHLVHWNTKYGDFGKAVQQPDGLAVLGIFLKVGSAKPGLQKVVDVLDSIKTKGKSADFTNFDPRGLLPESLDYWTYPGS
LTTPPLLECVTWIVLKEPISVSSEQVLKFRKLNFNGEGEPEELMVDNWRPAQPLKNRQIKASFKGAPGFSSISAHHHHHHHHHH

AChE-SNAP-Pro30-CLIP-HCA pDisplay

METDTLLLWVLLLWVPGSTGDYPYDVPDYAGAQPAELKVSTQTGSVRGLSLPVLDGHVSAFLGIPFAEPPLGRMRFLRPE
PVKPWQHVLDATSYKPACYQMVDTSYPGFQGTEMWNPNRGMSEDCLYLNIWVPSPRPKDAPVLVWIYGGGFYSGAASLDVYDGR
FLTYTQNVILVSLSYRVGAFGFLGLPGSPEAPGNMGLLDQRLALQWIQNNIHPFGGNPRAVTVFGEAAGAASVGMHLLSTQSRTLFQ
RAILQSGGPNAPWATVTPAESRRRAALLGKQLGCHFNNDSELVSCLRSKNPQELIDEEWSVLPYKSIFRFPFVPVIDGDFFPDTPEAM
LSSGNFKETQVLLGVVKDEGSYFLIYGLPGFSKDNESLISRADFLEGVRMSVPHANDIATDAVVLQYTDWQDQDNREKNREALDDIV
GDHNVICPVVQFANDYAKRNSKVYAYLFDHRASNLLWPPWMGVPHGYEIEFVFGLPLNDSLNYTPQEKELSRRMMRYWANFARTG
NPTDPADKSGAWPTYTASQPQYVQLNTQPLATQPSLRAQICAFWNHFLPKLLNATCTGGSGGSGGSGGSMDKDCEMKRTTLDSPL
GKLELSGCEQGLHEIIFLGKGTSAADAVEVPAPAAVLGGPEPLMQATAWLNAYFHQPEAIEEFPVPALHHPVFQQESFTRQVLWKLLK
VVKFGEVISYSHLAALAGNPAATAAVKTALSGNPVPILIPCHRVVQGDLDVGGYEGGLAVKEWLLAHEGHRLGKPGLGGRPPPPPPP
PPPPPPPPPPPPPPPPPPPPPPPGGRMDKDCEMKRTTLDSPLGKLELSGCEQGLHKIIFLGKGTSAADAVEVPAPAAVLGGPEPLIQA
TAWLNAYFHQPEAIEEFPVPALHHPVFQQESFTRQVLWKLLKVVKFGEVISESHLAALVGNPAATAAVNTALDGNPVPILIPCHRVVQ
GDSDVGPYLGGLAVKEWLLAHEGHRLGKPGLGLEMSHHWGYGKHNGPEHWHKDFPIAKGERQSPVDIDTHTAKYDPSLKPLSVSY
DQATSLRILNNGHAFNVEFDDSQDKAVLKGGPLDGTYRLIQFHFHWGSLDGQGSEHTVDKKKYAAELHLVHWNTKYGDFGKAVQQ
PDGLAVLGIFLKVGSAKPGLQKVVDVLDSIKTKGKSADFTNFDPRGLLPESLDYWTYPGSLTTPPLLECVTWIVLKEPISVSSEQVLKF
RKLNFNGEGEPEELMVDNWRPAQPLKNRQIKASFKVDEQKLISEEDLNAVGQDTQEVIVVPHSLPFKVVVISAILALVVLTIISLIILIMLW
QKKPR

SUPPLEMENTARY DISCUSSION: Parameters affecting the switching equilibrium

The equilibrium of opening and closing of the CLASH constructs depends on the affinity of the two binding sites of the tethered ligand for their respective targets and on the effective molarity of the intramolecular ligand for the reporter protein. The effective molarity (M_{eff}) for similar biosensor constructs was estimated to be around 100 µM **Error! Reference source not found.**, which we consider as a good estimate for the CLASH constructs.

In the absence of effector molecule, the tethered ligand should bind intramolecularly to its cognate receptor. If we stipulate that the CLASH construct should be to more than 90% in its closed conformation, we can calculate the minimal dissociation constant for the tethered ligand (K_{d1}) for the reporter protein as at most one tenth of the effective molarity, and thus around 10 µM. The amount of the effector protein to obtain half of the activation of the reporter depends on the K_{d2} for its respective ligand used in the CLASH, and can be estimated from the formula

M_{eff} K_{d2}/K_{d1}. For example if K_{d1} is 10 μM, a K_{d2} of 10 nM results in half-activation of the reporter at 100 nM effector concentration.

CLASH-AChE/HCA has two binding proteins in the same fusion protein. In the presence of saturating concentrations of the small molecule effector tacrine we want near-complete intramolecular binding of the sulfonamide moiety to HCA, i.e. that at least 90% of *CLASH*-AChE/HCA is in the HCA-bound state. Assuming again a M_{eff} of 100 μM, K_{d1}, i.e. the K_d of the untethered ligand towards HCA, has to be 10 μM or lower. On the other hand, in absence of tacrine we want near-complete binding of the tethered ligand to AChE, defined as 90% of *CLASH*-AChE/HCA being in its AChE-bound state. Assuming that the effective molarities for the HCA-ligand and for the AChE-ligand are similar, K_{d2} should be at least 10-fold lower than K_{d1}, namely 1 μM or lower. In such conditions the minimum amount of small molecule effector to obtain half-transition depends on the affinity of the effector tacrine for AChE (K_{dE}), and can be estimated by M_{eff} K_{dE}/K_{d2}.

The kinetics of the CLASH constructs is mainly governed by the unbinding of the intramolecular ligand from the respective receptor protein, while closing is driven by the unbinding of the effector protein, as was previously described for the similar semisynthetic biosensors SNIFITs [3]. In case of processes involving very slow off-rates, as for examples for the unbinding of biotin from streptavidin, the activation after the addition of the protein can be considered practically irreversible. On the contrary, in Figure 4 we demonstrated that *CLASH*-AChE/HCA is reversibly changing its conformation upon perfusion and removal of the effector tacrine. In this case the transition kinetics upon addition of tacrine can be estimated by the k_{off} of the tethered edrophonium from AChE; while upon removal of tacrine two mechanisms need to be considered: unbinding of the intramolecular sulfonamide from HCA and unbinding of tacrine from AChE; since the latter is the slower, the kinetic of the transition is governed by the k_{off} of tacrine from AChE.

SUPPLEMENTARY METHODS: Synthetic procedures

All reactions were carried out in oven-dried glassware under nitrogen atmosphere, unless stated otherwise. Molecular sieves 4Å (MS4A) were purchased from Sigma Aldrich and activated by heating at 120°C for at least 24 hours. Chemicals were purchased from Sigma-Aldrich, Fisher Scientific, Merck, Alfa Aesar or Acros and used without further purification. Anhydrous solvents from Acros were used without further treatment. Flash column chromatography was performed with Merck silica gel (230-400 mesh). Reverse-phase analytical high-pressure liquid chromatography (analytical-HPLC) was run on a Dionex system equipped with a P680 pump, an ASI 100 automatic sample injector and an UltiMate 3000 diode array detector using a Waters symmetry C18 column (5μm, 3.9 x 150 mm). Buffer A: 0.1% w/v TFA in H_2O Buffer B: acetonitrile; typical gradient was from 0% to 100% B within 15 minutes with 1 mL/min flow. Reverse-phase preparative high-pressure liquid chromatography (Prep-HPLC) was performed on a Dionex system equipped with an UltiMate 3000 pump and an UV D170U UV-Vis detector on a Waters SunFire Prep C18 OBD 5 μm 19×150 mm Column; a typical gradient was from 0% to 100% B within 22 minutes with 5 mL/min flow. LC-MS for the final labeling compounds was performed on a Shimadzu MS2020

connected to a Nexera UHPLC system equipped with a Waters ACQUITY UPLC BEH C18 1.7 µm 2.1x50 mm column. Buffer A: 0.05% HCOOH in H_2O Buffer B: 0.05% HCOOH in acetonitrile. Analytical gradient was from 10% to 90% B within 5 min with 0.5 ml/min flow. Thin-layer chromatography (TLC) was performed on Silica gel 60 F254 aluminum foils (Merck). Proton and carbon nuclear magnetic resonance (NMR) spectra were recorded at room temperature on a Bruker Avance-III 400 or on a Bruker DRX-600 equipped with a cryoprobe, with chemical shifts (δ) reported in ppm relative to the solvent residual signals. $CDCl_3$: δ_H 7.26ppm, δ_C 77.16 ppm; DMSO-d_6: δ_H 2.5 ppm, δ_C 39.5 ppm; CD_3OD: δ_H 3.31 ppm, δ_C 49.0 ppm. Coupling constants are reported in Hz. Compounds for which the synthesis scale did not allow the acquisition of a carbon NMR spectrum are characterized by proton NMR and high resolution mass spectrometry. Mass spectra were recorded on a Thermo Finnigan TSQ 7000. Solvent and reagent abbreviations: DMSO – dimethylsulfoxyde, DMF – dimethylformamide, DCM – dichloromethane, EtOAc – ethyl acetate, DIEA – diisopropylethylamine, MS4A – molecular sieve 4 Å, TFA – trifluoroacetic acid, DBU – 1,8-Diazabicyclo[5.4.0]undec-7-ene, Et_2O – diethylether.
Suppliers of drugs and derivatives used for titrations: methotrexate (Applichem), topiramate (TCI), tacrolimus (Fluorochem), sirolimus (Sigma-Aldrich), cyclosporin A (Fluorochem), digoxin (Fluka), 7-hydroxy methotrexate (Santa Cruz Biotechnology), 4-amino-4-deoxy-N-methylpteroic acid (DAMPA) (Sigma-Aldrich).

Due to the very high sensitivity of the luminescent sensor proteins used, very small amounts (< 0.5 mg) of the final labeling compounds **1**-**4** were needed. Please note that we did not obtain interpretable ^{13}C-NMR spectra for compounds (**1**-**4**). The reason for that is the small scale of our syntheses combined with the large molecular weight of final products **1**-**4**, all in the range of 1.5-2.5k daltons. Given that compounds **1**-**4** were prepared from fully characterized building blocks using simple and well-known chemical reactions, the reported HRMS and ^{1}H-NMR data of compounds **1**-**4** in our opinion provide sufficient characterization.

General Procedure 1 – Amide bond formation
Amide bond formation was performed by activation of the respective carboxylic acid with O-(N-Succinimidyl)-N,N,N,N'-tetramethyluronium tetrafluoroborate (TSTU) or N,N,N',N'-Tetramethyl-O-(1H-benzotriazol-1-yl)uronium hexafluorophosphate (HBTU) in the presence of diisopropylethylamine (DIEA) as base in anhydrous DMSO at room temperature. In a standard coupling reaction, the carboxylic acid, the coupling agent (TSTU or HBTU) and the amine to be coupled were dissolved in separate flasks in anhydrous DMSO to a final concentration of 10 to 500 mM. 1 eq of the carboxylic acid solution was treated with 2.5 eq DIEA, followed by 1.2 eq of the coupling agent solution and stirred at room temperature. Activated ester formation was checked by TLC or analytical HPLC and, when complete, 1-3 eq of the amine solution was added. The reaction was further stirred at room temperature until the activated ester was completely consumed. The reaction was then quenched by addition of water and acidified with 3 eq acetic acid, before Prep-HPLC purification.

General Procedure 2 – Phenol alkylation

A typical reaction for alkylating a phenolic group was performed dissolving 1 eq of phenol in anhydrous DMF to a final concentration of 10-100 mM, followed by 1 eq of the alkyl electrophile, 2 eq of anhydrous potassium carbonate and an excess of MS4A. The suspension was vigorously stirred at 55°C until the reaction was judged complete by analytical-HPLC or TLC.

General Procedure 3 – Boc deprotection

The tert-butylcarbamate-protected compound was dissolved in an excess of TFA and stirred at room temperature. When the protecting group was completely removed as judged by analytical-HPLC, the excess of solvent and side products were removed at reduced pressure.

General Procedure 4– Fmoc deprotection

The 9-Fluorenylmethylcarbamate -protected compound was dissolved in acetonitrile and 2.5 eq of DBU were added. The solution was stirred until analytical HPLC indicated complete protecting group removal, and then acidified with 3 eq of glacial acetic acid. The solution was concentrated at reduced pressure and purified by preparative HPLC.

General Procedure 5 – Cy3 derivatives preparation

Cy3 derivative synthesis was performed using a modification of *General Procedure 1* using TSTU as coupling agent in a 2-step one-pot reaction, starting from the previously described bis-carboxy-Cy3 derivative **23** [30] and O^6-benzylguanine (BG) derivative **6** [5]. A 15 mM solution of Compound **23** (1.5 µmol) in anhydrous DMSO was treated with 2 eq of DIEA, 2 eq of TSTU and 1 eq of Compound **6** and shaken at room temperature for 30 min. 2 additional equivalents of DIEA were added, followed by 1.1 eq of the appropriate amino-derivative. The reaction was shaken for further 30 min at room temperature, and then quenched with 3 volumes of H_2O. The solution was then purified by preparative HPLC to give the product with typical yields of about 20%.

General Procedure 6 – Click chemistry

10 mM solution of alkyne was mixed with 1 eq of the appropriate azido-derivative, followed by 0.2 eq of Tris[(1-benzyl-1H-1,2,3-triazol-4-yl)methyl]amine, 0.2 eq of 20 mM copper sulfate solution in water and 1 eq of 100 mM sodium ascorbate solution in water. The clear solution was stirred at room temperature until the reaction was complete as judged by analytical-HPLC. The reaction was then purified by preparative HPLC. Yields for this procedure were generally around 90%.

Turn-on luciferase labeling compound

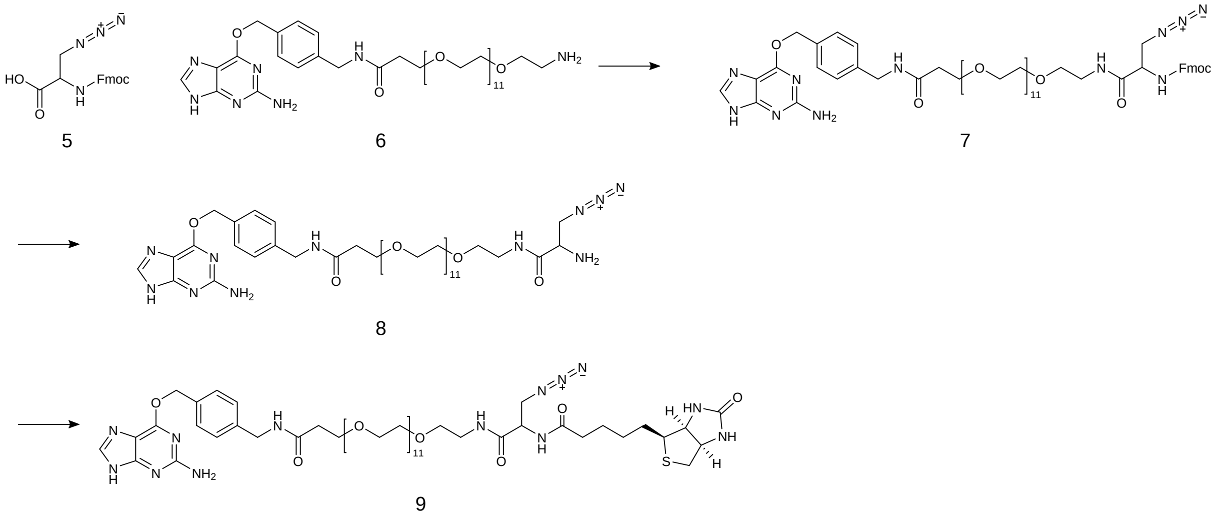

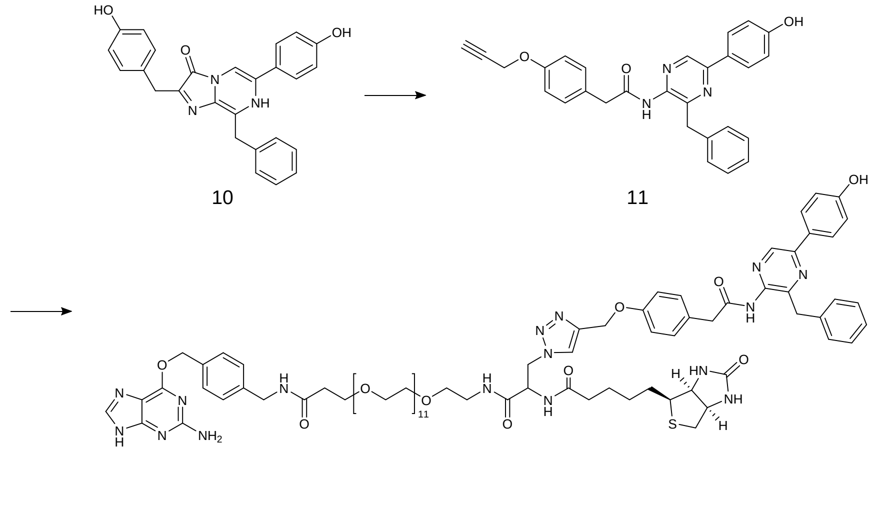

1

BG-azide/amine (8) Fmoc-L-Dap(N3)-OH **5** (20 mg, 52 mmol) was reacted with Compound **6** according to General Procedure 1. The crude Compound **7** obtained was treated according to General Procedure 4 to remove the protecting group and the product was purified by preparative HPLC to afford Compound **8** as a colorless liquid (10 mg, 20% yield). [MS (ESI) 982.52 m/z expected, 982.50 m/z measured. ^{1}H NMR (400 MHz, DMSO) δ 8.66 (t, 1 H, J = 5.5 Hz), 8.51 (s, 1 H), 8.41 (m, 3 H), 7.50 (d, 2 H, J = 8.2 Hz), 7.30 (d, 2 H, J = 8.2 Hz), 5.55 (s, 2 H), 4.29 (d, 2 H, J = 5.9 Hz), 4.01 (s, 1 H), 3.78 (dd, 1 H, J = 13.4, 4.3 Hz), 3.72 (dd, 1 H, J = 13.4, 6.3 Hz), 3.64 (t, 2 H, J = 6.4 Hz), 3.44-3.55 (m, 46H), 3.30 (m, 2 H), 2.39 (t, 2 H, J = 6.3 Hz). ^{13}C NMR (101 MHz, MeOD) δ 128.7, 127.3, 70.1, 70.0, 69.9, 67.2, 66.9, 52.7, 52.5, 50.9, 50.7, 49.3, 45.1, 43.8, 42.3, 39.4, 39.0, 36.3, 31.7, 29.4, 29.2, 28.0, 27.7, 25.6, 23.3, 23.0]

BG-azide/Biotin (9) Biotin (4 mmol) and Compound **8** (4 mmol) were coupled according to General Procedure 1 with TSTU. The product was purified by preparative HPLC to afford Compound **9** as a colorless liquid (72% yield). [MS (ESI) 1208.60 m/z expected, 1208.58 m/z measured. ^{1}H NMR (400 MHz, DMSO) δ 8.10 (s, 1H), 7.53 (d, J = 7.9

Hz, 2H), 7.36 (d, J = 8.0 Hz, 2H), 5.62 (s, 2H), 4.60 (t, J = 6.1 Hz, 1H), 4.51 (dd, J = 8.0, 4.8 Hz, 1H), 4.44 (s, 2H), 4.33 (m, 1H), 2.99 – 2.91 (m, 1H), 3.78 (t, J = 6.0 Hz, 2H), 3.71-3.54 (m, 50H) 2.74 (s, 1H), 2.71 (s, 1H), 2.68 (s, 14H), 2.52 (t, J = 6.0 Hz, 2H), 1.83 – 1.59 (m, 4H), 1.49 (q, J = 7.4 Hz, 5H), 1.31 (s, 6H). ^{13}C NMR (101 MHz, MeOD) δ 170.5, 168.8, 164.8, 128.9, 127.4, 70.1, 61.9, 60.3, 55.4, 39.7, 30.0, 27.9, 25.1, 24.3]

O-propargyl-Coelenteramide (11) Commercial coelenterazine **10** (2.0 mg, 4.72 µmol; from NanoLight Technology, Pinetop AZ) was treated according to General Procedure 2 with propargyl bromide (4.72 µmol) to afford Compound **11** as a pale yellow solid (0.6 mg, 28 % yield). [MS (ESI) 450.19 m/z expected, 450.15 m/z measured. ^{1}H NMR (400 MHz, MeOD) δ 8.78 (s, 1H), 8.06 (d, J = 8.8 Hz, 2H), 7.21 (m, 5H), 7.14 (d, J = 8.8 Hz, 2H), 7.06 (d, J = 6.8 Hz, 2H), 6.80 (d, J = 8.4 Hz, 2H), 4.83 (d, J = 2.4 Hz, 2H), 4.15 (s, 2H), 3.61 (s, 2H), 3.00 (t, J = 2.5 Hz, 1H). ^{13}C NMR (101 MHz, MeOD) δ 159.2, 156.4, 151.4, 149.7, 143.1, 136.9, 130.1, 128.9, 128.6, 128.0, 127.8, 126.0, 125.3, 120.0, 115.1, 115.0, 78.1, 75.7, 55.3, 41.8, 39.9]

BG-Coelenteramide/Biot (1) Compound **9** (82 nmol) was coupled via click chemistry to Compound **11** (82 nmol) according to General Procedure 6. The product was purified by preparative HPLC to afford Compound **1** as a brownish liquid (62% yield). [MS (ESI) 829.38 m/z M^{2+} expected, 829.58 m/z M2+ measured. ^{1}H NMR (400 MHz, DMSO) δ 8.41 (s, 1 H), 8.20 (d, 1 H, J = 0.6 Hz), 8.07 (s, 1 H), 7.93 (d, 1 H, J = 8.8 Hz), 7.45 (d, 1 H, J = 8.0 Hz), 7.33 (m, 2 H), 7.27 (m, 2 H), 7.14 (d, 1 H, J = 8.6 Hz), 7.01 (d, 1 H, J = 8.6 Hz), 6.42 (m, 2 H), 6.35 (m, 1 H), 5.47 (s, 1 H), 5.11 (s, 2 H), 4.72 (m, 3 H), 4.27 (m, 2 H), 4.07 (s, 2 H), 3.90 (s, 2 H), 3.62 (t, 2 H, J = 6.6 Hz), 3.54 (m, 2 H), 3.48 (m, 2 H), 3.38 (m, 20 H), 3.25 (s, 10 H), 3.17 (m, 4 H), 2.40 (m, 2 H), 1.48 (m, 4 H), 1.23 (s, 2 H).]

Benzenesulfonamide/Biotin dual ligand

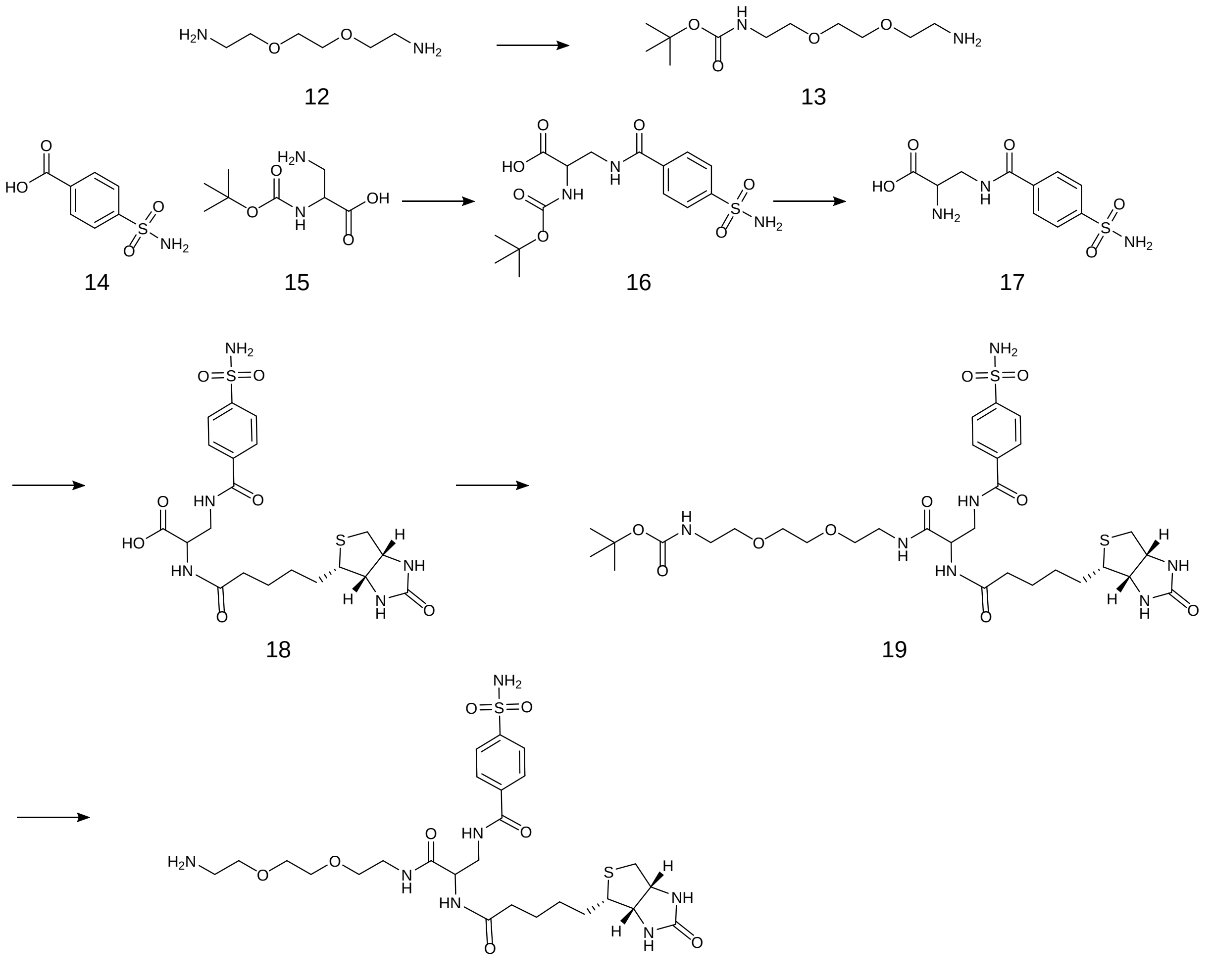

20

N–Boc–2,2'–(ethylenedioxy)diethylamine (**13**) 2,2'–(ethylenedioxy)diethylamine **12** (30 mmol) was dissolved in 30 ml DCM in a 2–necked flask. Ditert–butyl dicarbonate (5.5 mmol) was dissolved in 20 ml DCM, poured in a dropping funnel and added drop wise over 20 min. After 2 h the solution was extracted 2×20 ml NaCl (sat) + 1×20 ml H2O. The organics were dried over Na_2SO_4, filtered and concentrated. The crude transparent and highly viscous liquid was lyophilized and used without any further purification (1.25 g, 77%). [TLC–silica: Rf(EtOAc/EtOH/H2O 5:3:2)= 0.21. HRMS (ESI):249 m/z. ^{1}H NMR $CDCl_3$ δ: 3.65 (m, 4 H), 3.52 (m, 4 H), 3.35 (m, 2 H), 2.90 (t, J = 5.2 Hz, 1 H), 1.46 (s, 9 H). ^{13}C NMR (101 MHz, $CDCl_3$) δ: 156.0, 79.19, 73.5, 70.2, 53.4, 41.8, 40.4, 28.4].

2-(((tert-butoxy)carbonyl)amino)-3-((4-sulfamoylphenyl)formamido)propanoic acid (16) 4-sulfamoylbenzoic acid **14** (30 mg, 150 mmol), was coupled with (S)-2-[(tert-butoxycarbonyl)amino]-3-aminopropionic acid **15** (30 mg, 150 mmol) according to General Procedure 1 using TSTU. The crude product was purified by preparative HPLC to afford Compound **16** as a colorless liquid (24 mg, 62% yield). [^{1}H NMR (400 MHz, DMSO) δ 12.66 (m, 1 H), 8.69 (t, 1 H, J = 5.6 Hz), 7.93 (s, 2 H), 7.89 (m, 2 H), 7.49 (s, 2 H), 7.13 (d, 1 H, J = 8.2 Hz), 4.19 (m, 1 H), 3.57 (dd, 2 H, J = 6.4, 2.7 Hz),

1.36 (s, 9 H). ^{13}C NMR (101 MHz, MeOD) δ 172.4, 168.0, 156.6, 146.4, 137.4, 127.9, 127.8, 125.9, 79.4, 53.4, 41.19, 27.3]

2-amino-3-((4-sulfamoylphenyl)formamido)propanoic acid (17) Compound **16** (24 mg, 62 mmol) was treated according to General Procedure 3. The solvents were removed under reduced pressure to afford Compound **17** as a colorless liquid, which was used without any further purification (17 mg, 95% yield). [MS (ESI) 288.07 m/z expected, 288.08 m/z measured. ^{1}H NMR (400 MHz, DMSO) δ 8.92 (t, 1 H, J = 5.6 Hz), 8.34 (s, 2 H), 8.00 (m, 2 H), 7.91 (m, 2 H), 7.51 (s, 2 H), 4.11 (d, 1 H, J = 4.4 Hz), 3.74 (m, 2 H). ^{13}C NMR (101 MHz, DMSO) δ 169.7, 166.7, 147.0, 137.2, 128.6, 126.0, 52.6.]

2-(biotinylamino)-3-((4-sulfamoylphenyl)formamido)propanoic acid (18) Biotin (12 mg, 49 mmol) was coupled to Compound **17** (16 mg, 55 mmol) according to General Procedure 1 and purified by preparative HPLC to give Compound **18** as a white solid (15 mg, 59% yield) [MS (ESI) 514.14 m/z expected, 514.17 m/z measured. ^{1}H NMR (400 MHz, MeOD) δ 7.98 (m, 4H), 4.77 (m, 1H), 4.51 (m, 1H), 4.23 (m, 1H), 3.83 (m, 2H), 2.72 (m, 1H), 2.31 (m, 2H), 1.85-1.29 (m, 8H). ^{13}C NMR (101 MHz, MeOD) δ 174.8, 171.7, 168.0, 146.5, 137.4, 127.7, 125.9, 61.8, 60.2, 55.4, 52.3, 40.9, 39.6, 37.5, 35.1, 33.2, 33.2, 29.1, 28.0, 25.2]

N-(8-amino-3,6-dioxaoctyl)-2-(biotinylamino)-3-((4-sulfamoylphenyl) formamido) propanamide (20) Compound **18** (3.0 mg, 5.8 mmol) was activated using TSTU and coupled with Compound **13** (1.4 mg, 5.8 mmol) according to General Procedure 1. The reaction was purified by preparative HPLC to afford Compound **19** as a colorless liquid. The product was treated according to General Procedure 2 to give Compound **20** as a yellowish liquid (2.0 mg, 53% yield). [MS (ESI) 644.25 m/z expected, 644.42 m/z measured. ^{1}H NMR (400 MHz, DMSO) δ 8.62 (t, 1 H, J = 5.5 Hz), 7.99 (m, 2 H), 7.94 (m, 2 H), 7.88 (m, 2 H), 7.76 (s, 3 H), 7.50 (s, 2 H), 6.39 (m, 2 H), 4.48 (q, 1 H, J = 7.5 Hz), 4.29 (dd, 1 H, J = 7.4, 5.2 Hz), 4.07 (dd, 1 H, J = 7.3, 4.2 Hz), 3.55 (d, 3 H, J = 5.3 Hz), 3.50 (d, 6 H, J = 6.3 Hz), 3.36 (m, 3 H), 3.21 (m, 3 H), 2.98 (m, 3 H), 2.80 (dd, 1 H, J = 12.5, 4.9 Hz), 2.12 (m, 2 H), 1.55 (m, 1 H), 1.45 (m, 3 H), 1.27 (m, 2 H). ^{13}C NMR (101 MHz, MeOD) δ 127.8, 126.0, 69.9, 69.8, 69.0, 66.5, 61.8, 60.2, 55.9, 55.4, 53.2, 41.3, 39.6, 39.3, 39.0, 35.0, 28.0, 27.9, 25.2]

Trimethoprim/Biotin dual ligand

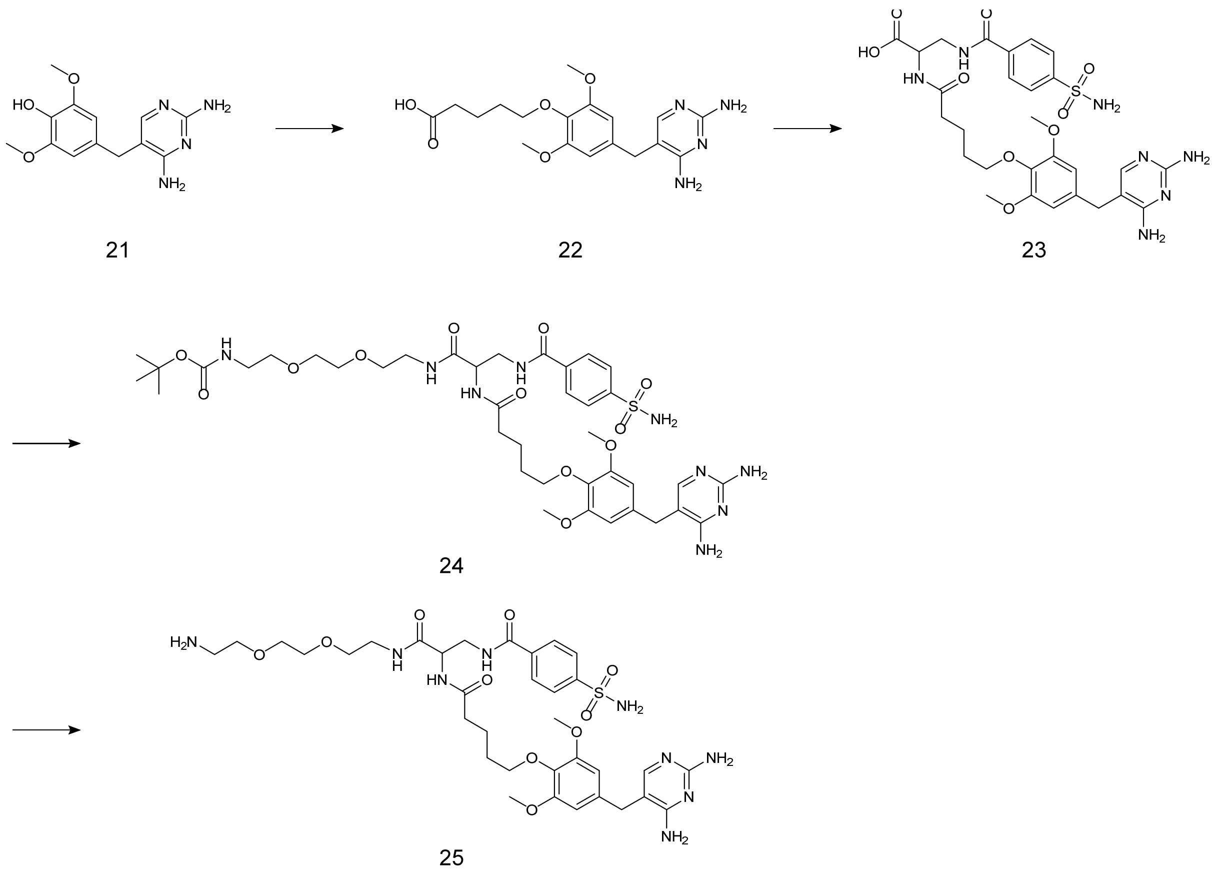

5-Trimethoprim-pentanoic acid (22) Compound **22** was produced according to General Procedure 2 starting from 4-demethyl trimethoprim **21** (0.12 mmol), prepared as previously described [1], and methyl 5-bromo-pentanoate (0.12 mmol). The reaction was diluted in 15 ml EtOAc and filtered the insoluble. The organic phase was washed with 3×15 ml H_2O and the solvent removed at reduced pressure. The residue was taken up in 1 ml MeOH and 1 ml 1 M aqueous NaOH were added to hydrolyze the methyl ester and the solution stirred for 1 hour at room temperature. The reaction was acidified with AcOH and purified by preparative HPLC to afford Compound **22** as a yellowish solid (4.0 mg, 20% yield). [MS (ESI) 377.18 m/z expected, 377.25 m/z measured. ^{1}H NMR (400 MHz, DMSO) δ 11.92 (s, 1 H), 8.29 (s, 1 H), 7.75 (s, 1 H), 7.61 (s, 2 H), 7.43 (s, 1 H), 6.60 (s, 2 H), 3.78 (t, 2 H, J = 5.9 Hz), 3.72 (s, 6 H), 3.63 (s, 1 H), 3.58 (s, 2 H), 2.26 (t, 2 H, J = 6.9 Hz), 1.63 (m, 4 H). ^{13}C NMR (101 MHz, DMSO) δ 154.7, 153.5, 140.2, 133.3, 109.4, 106.7, 72.3, 56.4, 36.1, 33.7, 32.6, 29.5, 21.6.]

2-(5-O-trimethoprim-pentanoylamino)-3-((4-sulfamoylphenyl)formamido)propanoic acid (23) Compound **22** (1.5 mg, 4.0 mmol) was coupled with Compound **17** (1.2 mg, 4.0 mmol) according to General Procedure 1 using TSTU. The reaction was purified by preparative HPLC to afford Compound **23** as a yellow liquid, which was used in the following step without further characterization. (1.0 mg, 39% yield). [MS (ESI) 646.23 m/z expected, 646.33 m/z measured. ^{1}H NMR (400 MHz, MeOD) δ 7.95 (m, 4H), 7.24 (s, 1H), 6.57 (s, 2H), 4.77 (m, 1H), 3.90 (m, 2H), 3.80 (s,

6H), 3.68 (s, 2H), 2.37 (t, 2H, J = 7.36 Hz), 1.82 (m, 2H), 1.71 (m, 2H) ^{13}C NMR (101 MHz, MeOD) δ 153.7, 139.0, 137.4, 135.8, 127.7, 125.9, 105.9, 72.4, 55.2, 52.3, 40.9, 37.5, 35.1, 32.5, 29.3, 29.1, 22.1]

N-(8-amino-3,6-dioxaoctyl)-2-(biotinylamino)-3-((4-sulfamoylphenyl) formamido) propanamide (25) Compound **23** (0.5 mg, 0.8 mmol) was activated using TSTU and coupled with Compound **13** (0.2 mg, 0.8 mmol) according to General Procedure 1. The reaction was purified by preparative HPLC to afford Compound **24** as a colorless liquid. The product was treated according to General Procedure 2 to give Compound **25** as a yellowish liquid (0.3 mg, 52% yield). [MS (ESI) 776.34 m/z expected, 776.22 m/z measured. ^{1}H NMR (400 MHz, DMSO) δ 11.80 (m, 1 H), 8.62 (d, 1 H, J = 4.8 Hz), 8.30 (s, 1 H), 8.00 (d, 2 H, J = 4.8 Hz), 7.94 (m, 2 H), 7.88 (m, 2 H), 7.75 (s, 3 H), 7.58 (s, 1 H), 7.49 (s, 2 H), 7.42 (s, 1 H), 6.60 (s, 1 H), 4.48 (m, 2 H), 3.71 (s, 2 H), 3.56 (s, 4 H), 3.16 (s, 8 H), 2.97 (d, 2 H, J = 4.6 Hz), 2.19 (m, 2 H), 1.60 (m, 4 H). ^{13}C NMR (101 MHz, MeOD) δ 174.9, 170.8, 169.0, 164.9, 154.7, 153.6, 137.2, 132.4, 127.7, 125.9, 120.0, 109.6, 106.7, 72.9, 69.9, 69.0, 66.9, 55.2, 53.4, 41.4, 39.3, 38.9, 35.11, 32.5, 29.1, 22.2]

BG-Cy3-Dual Ligand labeling compounds

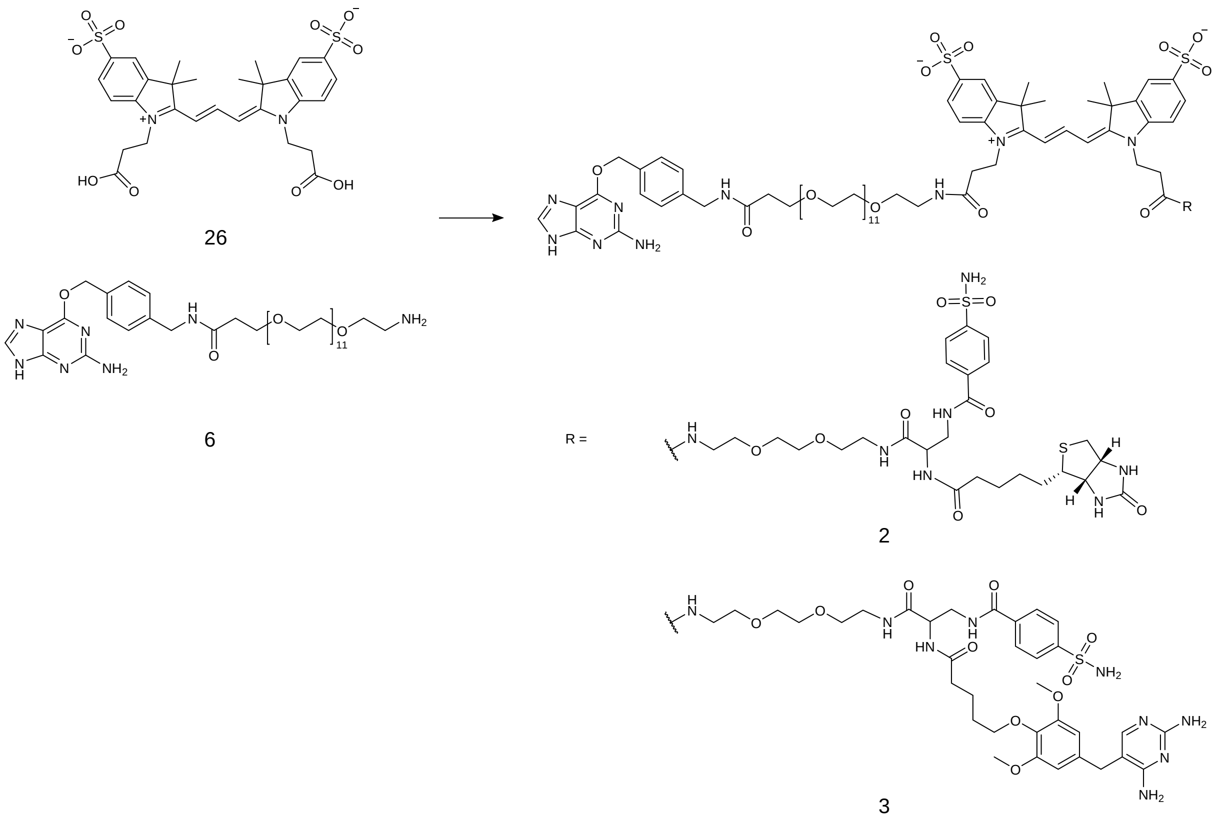

BG-Cy3-SA/biot (2) General Procedure 5 was adopted, coupling Compound **20** (0.06 mg, 28% yield). [MS (ESI) 1055.42 m/z M^{2+} expected, 1055.8 m/z M^{2+} measured. ^{1}H NMR (400 MHz, MeOD) δ 8.59 (t, J = 13.4 Hz, 1H), 8.39 (s, 1H), 7.99 (m, 6H), 7.93 (d, J = 8.7 Hz, 2H), 7.54 (d, J = 8.0 Hz, 2H), 7.45 (dd, J = 8.4, 4.9 Hz, 2H), 7.38 (d, J = 7.9 Hz, 2H), 6.55 (d, J = 13.4 Hz, 2H), 5.67 (s, 2H), 4.68 (q, J = 6.4 Hz, 1H), 4.50 (m, 5H), 4.25 (td, J = 9.0, 8.0, 4.5 Hz, 1H),

3.84 (p, J = 5.7 Hz, 2H), 3.77 (t, J = 5.9 Hz, 2H), 3.72 (d, J = 6.5 Hz, 2H), 3.66 (m, 2H), 3.58 (m, 46H), 3.10 (m, 2H), 2.92 (dd, J = 4.9, 2.7 Hz, 2H), 2.89 (dd, J = 5.0, 2.8 Hz, 2H), 2.78 (d, J = 6.6 Hz, 4H), 2.31 (dd, J = 8.4, 5.8 Hz, 2H), 1.81 (d, J = 2.5 Hz, 9H), 1.55 (dd, J = 13.7, 7.7 Hz, 2H), 1.26 (d, J = 7.1 Hz, 2H)]

BG-Cy3-SA/TMP (3) General Procedure 5 was adopted, coupling Compound **25** (0.04 mg, 25% yield). [MS (ESI) 1121.47 m/z M^{2+} expected, 1121.95 m/z M^{2+} measured. ^{1}H NMR (400 MHz, MeOD) δ 8.56 (t, J = 13.4 Hz, 1H), 8.37 (d, J = 6.4 Hz, 1H), 7.94 (m, 6H), 7.54 (m, 2H), 7.39 (m, 4H), 7.26 (s, 1H), 6.55 (m, 3H), 5.67 (d, J = 5.8 Hz, 4H), 4.68 (dd, J = 7.6, 5.4 Hz, 1H), 4.50 (s, 4H), 4.43 (d, J = 5.0 Hz, 3H), 4.36 (t, J = 6.2 Hz, 2H), 3.95 (t, J = 6.1 Hz, 1H), 3.77 (s, 5H), 3.60 (m, 40H), 2.76 (q, J = 6.1 Hz, 4H), 2.70 (t, J = 6.8 Hz, 1H), 2.58 (m, 1H), 2.52 (m, 3H), 1.79 (m, 14H), 1.49 (m, 1H).

BG-Cy5-Benzenesulfonamide/Edrophonium labeling compound

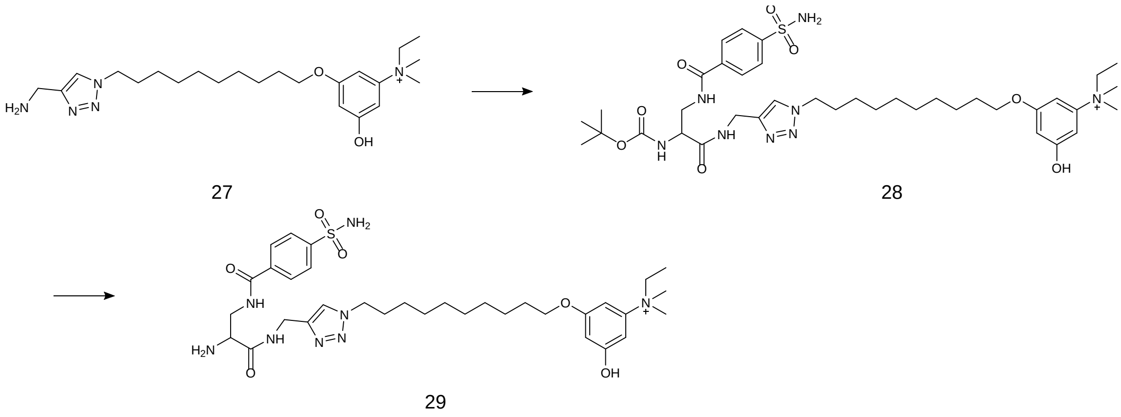

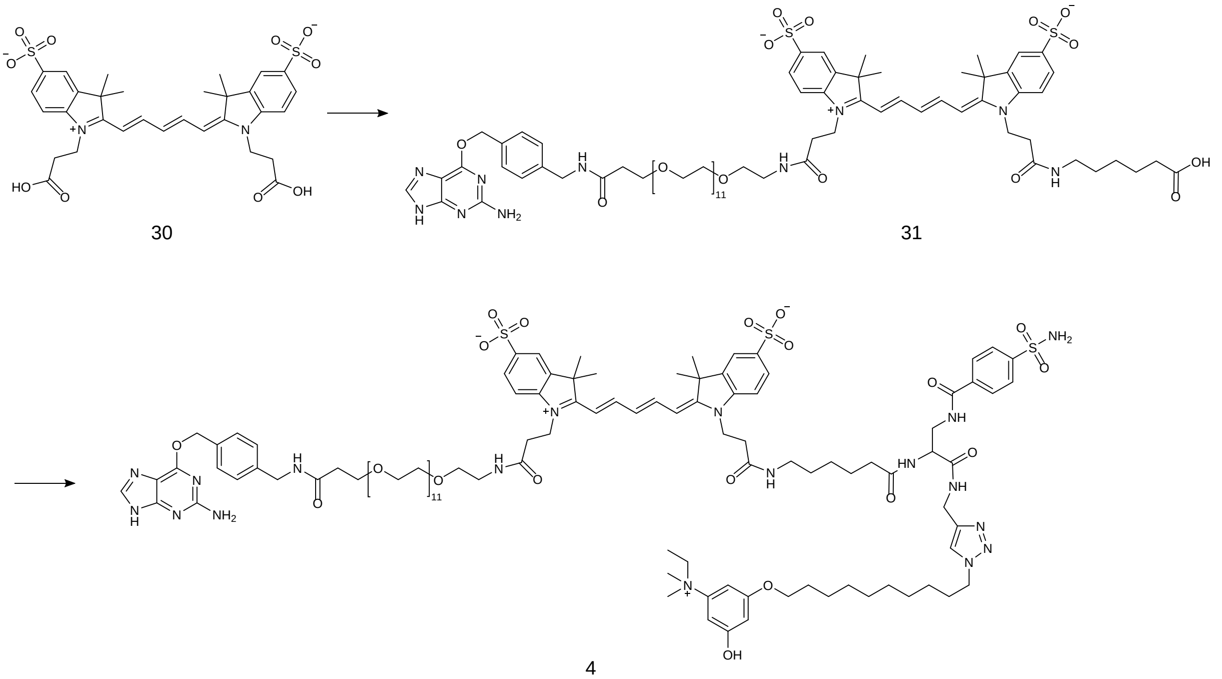

NH_2-EG_2-Benzenesulfonamide/Edrophonium (29) The synthesis of the edrophonium derivative **27** was previously described [2]. Compound **27** and was coupled to Compound **17** according to General Procedure 1. The crude Compound **28** was treated according to General Procedure 3, followed by purification by preparative HPLC to give Compound **29** as a white solid (0.3 mg, 78 % yield). [MS (ESI) 687.36 m/z expected, 687.42 m/z measured. ^{1}H NMR (400 MHz, DMSO) δ 10.31 (s, 1 H), 9.02 (t, 1 H, J = 5.3 Hz), 8.80 (m, 1 H), 8.27 (m, 3 H), 7.96 (m, 2 H), 7.90 (m, 3 H), 7.51 (s, 2 H), 6.83 (s, 1 H), 6.73 (s, 1 H), 6.49 (s, 1 H), 4.39 (m, 2 H), 4.25 (t, 3 H, J = 7.1 Hz), 3.95 (t, 4 H, J = 6.2 Hz), 3.83 (m, 3 H), 1.72 (m, 4 H), 1.31 (m, 12 H), 0.97 (t, 2 H, J = 6.9 Hz). ^{13}C NMR (101 MHz, MeOD) δ 171.5, 168.1, 161.58, 160.2, 146.5, 145.6, 144.8, 137.2, 127.7, 125.9, 122.7, 120.0, 102.1, 100.1, 98.9, 68.3, 64.5, 54.9, 53.0, 50.0, 41.4, 34.3, 29.8, 29.1, 29.0, 28.9, 28.8, 28.6, 27.3, 26.0, 25.7, 7.7]

BG-Cy5-C_5H_{10}-COOH (31) A modification of General Procedure 5 was adopted, using bis-carboxy-Cy5 derivative **30** that was previously described [3]. Compound **30** was sequentially coupled to Compound **6** and to 6-aminohexanoic acid. The reaction was purified by preparative HPLC to afford Compound **31** as a dark blue solid (0.34 mg, 52 % yield). [MS (ESI) 812.36 m/z M^{2+} expected, 812.65 m/z M^{2+} measured. ^{1}H NMR (400 MHz, MeOD) δ 8.47 (s, 0H), 8.36 (t, *J* = 13.0 Hz, 1H), 7.91 (m, 2H), 7.54 (d, *J* = 8.2 Hz, 1H), 7.39 (dd, *J* = 8.2, 3.4 Hz, 2H), 6.70 (t, *J* = 12 Hz, 1H), 6.43 (dd, *J* = 13.5, 5.0 Hz, 2H), 5.69 (s, 1H), 4.44 (m, 3H), 3.77 (t, *J* = 5.6 Hz, 1H), 3.60 (m, 24H), 3.39 (m, 39H), 3.30 (m, 4H), 3.11 (m, 2H), 2.71 (m, 3H), 2.52 (td, *J* = 5.9, 3.5 Hz, 2H), 2.26 (t, *J* = 7.4 Hz, 2H), 1.78 (s, 6H), 1.57 (m, 2H), 1.43 (m, 2H), 1.30 (m, 2H). ^{13}C NMR (101 MHz, MeOD) δ 175.87, 174.23, 172.70, 170.66, 159.53, 158.57, 155.06, 153.05, 151.81, 143.15, 143.08, 142.71, 142.18, 142.14, 141.69, 141.20, 141.13, 139.71, 133.68, 128.95, 127.35, 127.11, 126.77, 126.54, 119.98, 110.53, 110.37, 104.40, 70.14, 70.08, 70.04, 69.97, 69.80, 69.68, 68.84, 66.89, 49.31, 42.26, 40.64, 40.62, 39.22, 39.13, 36.39, 36.36, 28.64, 28.60, 26.50, 26.10, 26.04, 25.08, 24.96, 24.30, 24.25, 24.01.]

BG-Cy5-SA/Edro (4) Compound **29** (34 nmol) and Compound **31** (40 nmol) were coupled according to General Procedure 1 using TSTU. The crude product was purified by preparative HPLC to afford Compound **4** as a dark blue solid (0.05 mg, 19 % yield). [MS (ESI 1146.54 m/z M^{2+} expected, 1147.05 m/z M^{2+} measured. ^{1}H NMR (400 MHz, MeOD) δ 8.37 (m, 1H), 8.25 (s, 1H), 8.01 (s, 1H), 7.90 (m, 6H), 7.53 (d, J = 8.0 Hz, 2H), 7.38 (m, 4H), 7.31 (s, 1H), 6.81 (s, 1H), 6.74 (s, 1H), 6.71 (s, 1H), 6.56 (t, J = 1.9 Hz, 1H), 6.42 (m, 2H), 5.64 (s, 2H), 4.45 (m, 8H), 4.35 (t, J = 6.9 Hz, 1H), 4.01 (t, J = 6.3 Hz, 2H), 3.91 (q, J = 7.2 Hz, 2H), 3.77 (td, J = 5.9, 3.5 Hz, 3H), 3.60 (m, 55H), 3.03 (m, 1H), 2.72 (m, 4H), 2.51 (td, J = 6.0, 3.0 Hz, 3H), 2.30 (t, J = 7.4 Hz, 1H), 1.88 (p, J = 7.2 Hz, 2H), 1.75 (m, 12H), 1.30 (m, 20H), 1.15 (s, 3H)]

SUPPLEMENTARY REFERENCES

[1] Jing, V., Cornish, V.G. A Fluorogenic TMP-Tag for High Signal-to-Background Intracellular Live Cell Imaging. *ACS Chemical Biology* **8** 1704-1712 (2013).

[2] Schena, A., Johnsson, K. Sensing acetylcholine and anticholinesterase compounds. *Angew. Chem. Int. Ed.* **53** 1302-1305 (2014).

[3] Brun, M.A., Tan, K.T., Nakata, E., Hinner, M.J., Johnsson, K. Semisynthetic fluorescent sensor proteins based on self-labeling protein tags. *Journal of the American Chemical Society* **131** 5873-84 (2009).

[4] Krishnamurthy, V. M., Semetey, V., Bracher, P.J., Shen N., Whitesides, G.M. Dependence of effective molarity on linker length for an intramolecular protein-ligand system. *Journal of the American Chemical Society*, **129**, 1312-1320 (2007).

[5] Griss, R., Schena, A., Reymond, L., Patiny, L., Werner, D., Tinberg, C.E., Baker, D., Johnsson, K. Bioluminescent sensor proteins for point-of-care therapeutic drug monitoring. *Nat. Chem. Biol.* **10**, 598-603 (2014).
